# Butyrate administration is not sufficient to improve immune reconstitution in antiretroviral-treated SIV-infected macaques

**DOI:** 10.1038/s41598-022-11122-x

**Published:** 2022-05-06

**Authors:** Alexandra M. Ortiz, Jennifer Simpson, Charlotte A. Langner, Phillip J. Baker, Cynthia Aguilar, Kelsie Brooks, Jacob K. Flynn, Carol L. Vinton, Andrew R. Rahmberg, Heather D. Hickman, Jason M. Brenchley

**Affiliations:** 1 Barrier Immunity Section, Laboratory of Viral Diseases, Division of Intramural Research, National Institute of Allergy and Infectious Diseases (NIAID), National Institutes of Health (NIH), Bethesda, MD 20892 USA; 2 Viral Immunity and Pathogenesis Unit, Laboratory of Clinical Immunology and Microbiology, NIAID, NIH, Bethesda, MD 20892 USA

**Keywords:** HIV infections, Mucosal immunology, Metagenomics

## Abstract

Defective gastrointestinal barrier function and, in turn, microbial translocation have been identified as significant contributors to persistent inflammation in antiretroviral (ARV)-treated people living with HIV. Metabolic supplementation of short-chain fatty acids (SCFAs), generally produced by the commensal microbiome, may improve these outcomes. Butyrate is a SCFA that is essential for the development and maintenance of intestinal immunity and has a known role in supporting epithelial integrity. Herein we assessed whether supplementation with the dietary supplement sodium butyrate would improve immune reconstitution and reduce inflammation in ARV-treated, simian immunodeficiency virus (SIV)-infected rhesus macaques. We demonstrate that butyrate supplementation does not significantly improve immune reconstitution, with no differences observed in systemic CD4+ T-cell frequencies, T-cell functionality or immune activation, microbial translocation, or transcriptional regulation. Our findings demonstrate that oral administration of sodium butyrate is insufficient to reduce persistent inflammation and microbial translocation in ARV-treated, SIV-infected macaques, suggesting that this therapeutic may not reduce co-morbidities and co-mortalities in treated people living with HIV.

## Introduction

Fully suppressive antiretroviral (ARV) therapy does not completely ameliorate inflammation in people living with HIV, with chronic inflammation a leading contributor to reduced life expectancy among these individuals^1^. Although time to treatment initiation, lifestyle, and environmental factors contribute to magnitude, increased inflammation is persistent and is associated with defined co-morbidities including hypertension, osteoporosis, hyperlipidemia, cancer, hepatitis, and renal disease^1,2^. Indicators of inflammation are varied, including circulating acute phase reactants and markers of coagulation (high-sensitivity C-reactive protein (CRP) and D-dimer), antimicrobial immunity (soluble CD14 (sCD14), soluble CD163, endogenous endotoxin-core antibody), and non-specific cytokine release (IL-6 and IL-18)^2^. Through persistent stimulation, chronic inflammation further contributes to a slow exhaustion of the adaptive lymphocyte population, resulting in poor vaccine responsiveness and susceptibility to opportunistic infections^2,3^. The design and implementation of adjunct therapies to address this inflammation will further improve the life expectancy and quality of life for people living with HIV.

Microbial translocation is a significant contributor to persistent inflammation and incomplete immune reconstitution in ARV-treated HIV infections^4^. In addition to the presence of circulating markers of antimicrobial immunity, translocation is characterized by the presence of circulating microbial products such as lipopolysaccharide (LPS), peptidoglycan, flagellin, and 16S or bacterial CpG DNAs^5^. Translocation can be visualized across the gastrointestinal epithelium, where breaches in epithelial integrity allow access to the underlying mucosa and into systemic circulation^6,7^. In the progressive SIV non-human primate model of HIV infection, administration of the LPS-binding compound sevelamer prevents plasma LPS and sCD14 accumulation and limits CRP and D-dimer elevation in acute, ARV-untreated infection^8^. Furthermore, the use of antibiotics cotrimoxazole and vancomycin have been shown to reduce, though not eliminate, adverse health effects in ARV-untreated HIV and SIV infection, respectively^9,10^. Though useful in treating the effects of translocation, the prolonged co-administration of antibiotics, sevelamer, and other pharmaceuticals is impractical in the setting of prolonged, non-curative, ARV therapy^11^.

The commensal microbiome is essential for the development and maintenance of the gastrointestinal epithelium and intestinal immunity in part through the production of diffusible metabolites, including SCFAs such as butyrate^12,13^. Within colonocytes, butyrate is an energy source and signals through peroxisome proliferator activated receptor gamma (PPARγ) to drive cellular energy metabolism towards *b*-oxidation, thereby limiting oxygen availability and restricting the growth of pathogenic facultative anaerobes^14^. Additionally, butyrate enhances histone H3 acetylation by inhibiting histone deacetylases (HDACs)^15–18^. The associated epigenetic modifications support the development of regulatory T-cells (T_regs_)^19–21^ and the production of IL-22 from T-helper 22 (T_H_22) cells and innate lymphoid cells (ILCs)^22^. In part through the activity of IL-22, butyrate contributes to tight junction formation, intestinal-stem-cell epithelial regeneration, mucin production, the physiologic production of reactive oxygen species, and antimicrobial peptide production^23–25^. A low-abundance of butyrate-producing taxa has been described in some HIV cohorts^26–34^ and supplementation of treated HIV and SIV infections with prebiotics or probiotics^31,35,36^ has been shown to reduce microbial translocation and immune activation, improve CD4+ T-cell frequencies, and improve thymic output or reduce lymphoid fibrosis. As such, butyrate supplementation alone has been proposed as a natural and inexpensive therapeutic to reduce inflammation and improve immune reconstitution in treated infections^30,37^.

Herein, we demonstrate that oral butyrate supplementation in ARV-treated, SIV-infected macaques does not significantly improve immune reconstitution. Supplemented animals do not show improved systemic CD4+ T-cell recovery, do not exhibit alterations in T-cell functionality, nor do they exhibit evidence of improved intestinal repair or microbial translocation. Our findings demonstrate that sodium butyrate is not sufficient to improve immunodeficiency over and above the effects of ARVs themselves.

## Results

### Butyrate supplementation does not improve viral suppression or CD4+ T-cell recovery in ARV-treated, SIV-infected macaques

**Figure 1:**
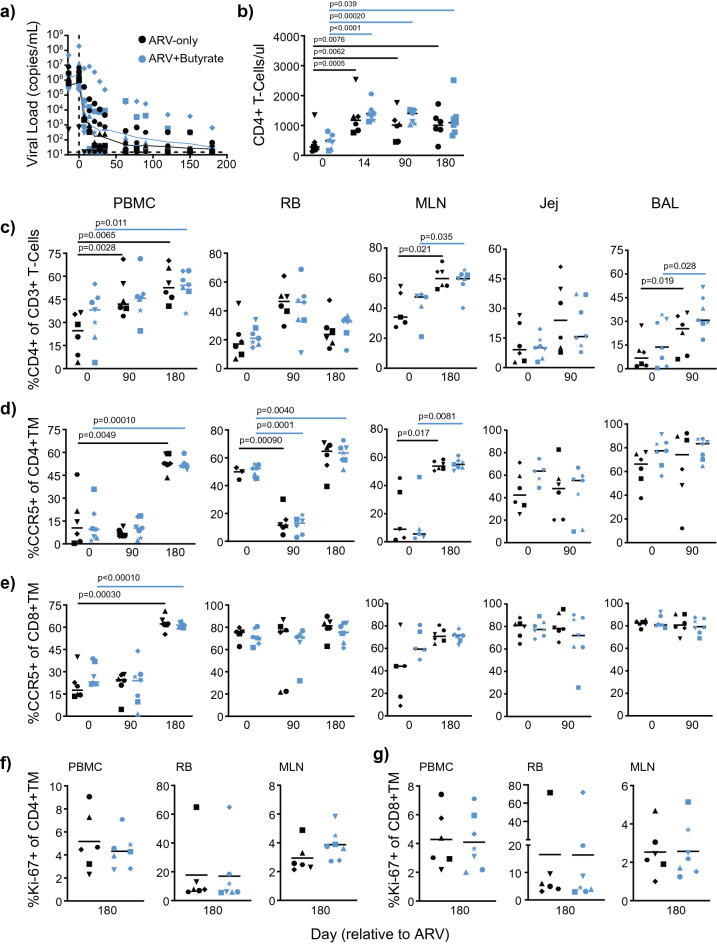
Butyrate supplementation of ARV treatment does not alter SIV suppression or target cell frequencies. (**a**) Longitudinal plasma SIV viral load in ARV-only (black) and ARV + butyrate (blue) animals, with individual animals denoted by individual symbols as identified in Table 1. (**b**) Circulating CD4+ T-cell count. (**c**–**e**) PBMC, RB, MLN, Jej, and BAL % CD4+ T-cells (**c**) and %CCR5 + CD4+ (**d**) and CD8 + (**e**) TM. (**f**, **g**) PBMC, RB, and MLN %Ki-67 + CD4+ (**f**) and CD8 + (**g**) TM. All timepoints are relative to the day of ARV initiation. Lines among datapoints represent means. Lines among *p*-values span timepoints considered significantly different, color-coded by treatment status. Significance methods as follows: Two-way ANOVA (**a**), unpaired and paired two-way *t*-tests as appropriate (**b**–**g**).

To investigate whether butyrate might improve immune reconstitution in treated SIV-infected Asian macaques, we initiated combination ARV treatment (emtricitabine, tenofovir disoproxil fumarate, and dolutegravir sodium) in chronically SIV-infected rhesus macaques 14 days prior to the administration of daily sodium butyrate (Table 1). ARV treatment was initiated prior to butyrate supplementation to ensure that suppression dynamics were comparable across the control and experimental groups. Following administration of ARVs, control (n = 6) and butyrate-supplemented (n = 7) animals were followed for 180 days, during which time blood and tissue lymphocytes were assessed for canonical markers of disease progression and immunological functionality. ARV initiation was associated with a rapid decline in plasma viral load in all animals (Fig. 1a). Although rates of viral decline and suppression varied across animals, viremia did not significantly differ between groups (two-way ANOVA). The exclusion of animals that did not effectively suppress viremia by study-end did not change the study outcome (data not shown). We next assessed the impact of butyrate administration on peripheral blood CD4+ T-cell counts and CD4+ T cell frequencies in tissues (Fig. 1b, c). As compared to baseline CD4+ T-cell values, ARV treatment with and without butyrate supplementation was associated with a significant recovery of CD4+ T-cells in the periphery, mesenteric lymph nodes (MLN), and bronchoalveolar lavage (BAL) by day 180 post-treatment and a trend towards recovery in rectal (RB) and jejunal (Jej) biopsies at day 90. However, no significant differences were noted by treatment status for any of the anatomic sites studied.

**Table 1:**
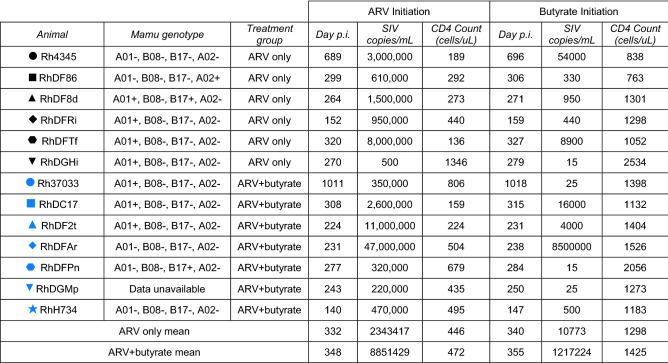
Study cohort characteristics at treatment initiation.

We next considered whether butyrate supplementation might influence the recovery of CD4+ memory T-cells (TM) expressing the SIV co-receptor CCR5. CCR5 frequencies increased from baseline to day 180 post-treatment in peripheral blood mononuclear cells (PBMCs) and the MLN for both groups. A significant increase was observed in RB for the butyrate-supplemented group only (Fig. 1d). For tissues sampled at day 90 post-treatment, CCR5 expression remained unchanged or decreased. To assess whether differences in CD4+ TM CCR5-expression might be mediated by residual viral replication, we also examined CCR5 expression on non-target, CD8 + TM. Here, a significant difference from baseline was noted only in PBMCs at day 180 post-treatment, with no significant differences observed between treatment groups (Fig. 1e).

Changes in CCR5 expression among tissue-based CD4+ T-cells may be the direct result of virus replication (or absence thereof) among CD4+ T-cells or, may be attributed to proliferation which might differ in the presence of butyrate. To assess these possibilities, we next measured Ki-67 expression among PBMC, RB, and MLN CD4+ and CD8 + TM on day 180 post-treatment (Fig. 1f, g). No differences were seen by treatment group, suggesting that the loss of direct infection was the main mechanism for CD4 recovery in both butyrate-untreated and -treated animals.

### Butyrate supplementation does not alter markers of mucosal homing or residency in ARV-treated, SIV-infected macaques

**Figure 2:**
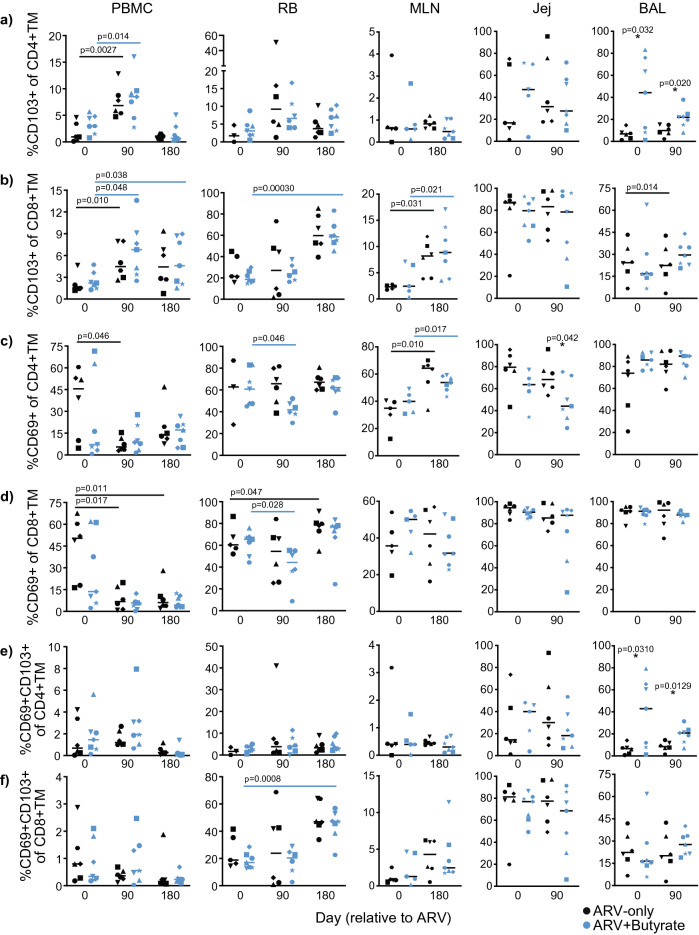
T-cell markers of intestinal homing and tissue residency remain unchanged with butyrate supplementation of ARV treatment. (**a**–**f**) PBMC, RB, MLN, Jej, and BAL %CD103 + (**a**, **b**) and CD69 + (**c**, **d**), CD69 + CD103 + (**e**, **f**) CD4+ (**a**, **c**, **e**) CD8 + (**b**, **d**, **f**) TM in ARV-only (black) and ARV + butyrate (blue) animals, with individual animals denoted by individual symbols as identified in Table 1. All timepoints are relative to the day of ARV initiation. Lines among datapoints represent means. Lines among *p*-values span timepoints considered significantly different, color-coded by treatment status. Asterisks denote timepoints where groups significantly differed. Significance assessed by unpaired and paired two-way *t* tests as appropriate.

We assessed whether butyrate-supplementation in ARV-treated, SIV-infected macaques might improve mucosal homing or retention by measuring expression of CD103, a highly expressed integrin in mucosal tissues^38^, and CD69, an often used marker of T-cell tissue residency^39^, in CD4+ and CD8 + TM across multiple tissue sites. Following ARV administration, T-cell CD103 expression significantly increased in multiple tissue sites as compared to baseline. Among CD4+ TM, CD103 expression increased only in PBMCs at day 90, irrespective of treatment group (Fig. 2a). A significant difference in CD4+ TM CD103 expression was noted between groups in the BAL at both baseline (two-way Student's *t* test, *p* = 0.032) and day 90 (*p* = 0.020); however, no longitudinal differences were noted for either group suggesting that these differences were independent of butyrate-supplementation. Among CD8 + TM, ARV-only animals exhibited an increase in CD103 expression from baseline to day 90 in PBMCs and to day 180 in the MLN and a decrease from day 0 to 90 in the BAL. CD8 + TM in butyrate-supplemented animals exhibited an increase from baseline to day 90 and 180 in PBMCs and to day 180 in RB and MLN (Fig. 2b). The significant increase in peripheral CD103 + memory T-cells in both groups at day 90 post-infection may reflect a redistribution of intestinal memory T-cells, contributing to the enhanced loss of CCR5-expressing cells (Fig. 1d) prior to complete viral suppression.

When considering CD69, we observed significant declines from baseline to day 90 in PBMC CD4+ and CD8 + TM from ARV-only treated animals and in RB CD4+ and CD8 + TM from butyrate-supplemented animals—this decline extended to day 180 among PBMC CD8 + TM only (Fig. 2c, d). In contrast, we observed increased CD69 expression from baseline to day 180 for both groups in MLN CD4+ TM and for ARV-only animals in RB CD8 + TM. Among Jej CD4+ TM, we observed a significant difference between groups at day 90 (*p* = 0.042), with significantly lower CD69 expression in butyrate-supplemented animals (Fig. 2c). As both groups exhibited a trend in declining CD69 expression among Jej CD4+ TM from day 0 to 90, this significance may indicate that butyrate-supplementation is accelerating this decline. We additionally considered that double-positive CD69 + CD103 + T-cells might differ in responsiveness to ARV therapy. Here differences were observed only among BAL CD4+ TM and RB CD8 + TM and reflected the changes observed in CD103 alone (Fig. 2e, f). Between group differences were noted in BAL CD4+ TM at both baseline (*p* = 0.0310) and day 90 post-treatment (*p* = 0.0129), suggesting that these differences were, in part, independent of butyrate supplementation. From day 0 to 180 post-treatment, a significant increase in CD69 + CD103 + was observed for ARV + butyrate RB CD8 + TM alone; however, no differences were noted between groups at day 180 post-treatment.

### Butyrate supplementation does not differentially affect T-cell functionality in ARV-treated, SIV-infected macaques

**Figure 3:**
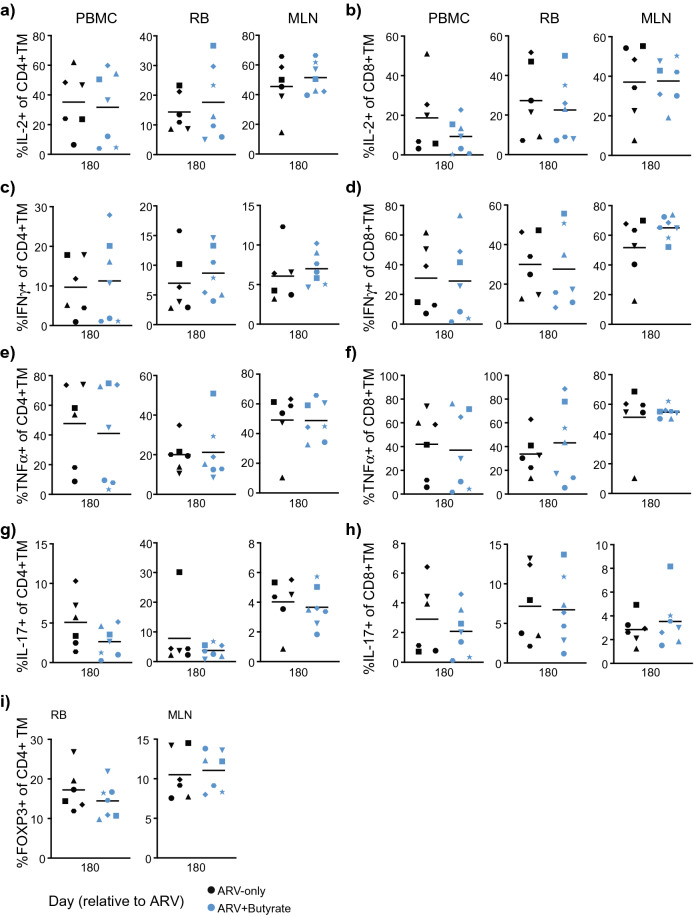
T-cell function does not improve with butyrate supplementation of ARV treatment. (**a**–**h)** PBMC, RB, and MLN %IL-2^+^ (**a**, **b**), IFNγ^+^ (**c**, **d**), TNFα^+^ (**e**, **f**), and IL-17 ^+^ (**g**, **h**) CD4^+^ (**a**, **c**, **e**, **g**) and CD8 ^+^ (**b**, **d**, **f**, **h**) TM in ARV-only (black) and ARV + butyrate (blue) animals, with individual animals denoted by individual symbols as identified in Table 1. (**i**) %FOXP3 + RB and MLN CD4+ TM. All timepoints are relative to the day of ARV initiation. Lines among datapoints represent means. Significance assessed by unpaired two-way *t* tests.

To investigate whether butyrate supplementation improved T-cell function, we assessed at day 180 post-treatment whether homeostatic proliferation may be altered by measuring IL-2, whether proinflammatory cytokine capacity was altered by measuring IFNg and TNFa, and whether antimicrobial function was altered by measuring IL-17. No significant differences were observed between treatment groups for either CD4+ or CD8 + TM isolated from PBMCs, RB, or MLN (Fig. 3a–h).

An imbalance of T_reg_ frequencies impedes immune reconstitution in treated HIV and SIV infections^40,41^. As butyrate signaling promotes the development of regulatory lymphocytes^19–21^, we considered that butyrate supplementation might specifically influence frequencies of intestinal T_regs_. When we investigated frequencies of RB and MLN CD4+ T_regs_ (FOXP3 + CD4+ TM) however, we saw no significant differences between groups (Fig. 3i). These results indicate that butyrate supplementation did not improve post-therapeutic T-cell function in SIV-infected macaques.

### Butyrate supplementation does not improve intestinal integrity nor microbial translocation in ARV-treated, SIV-infected macaques

**Figure 4:**
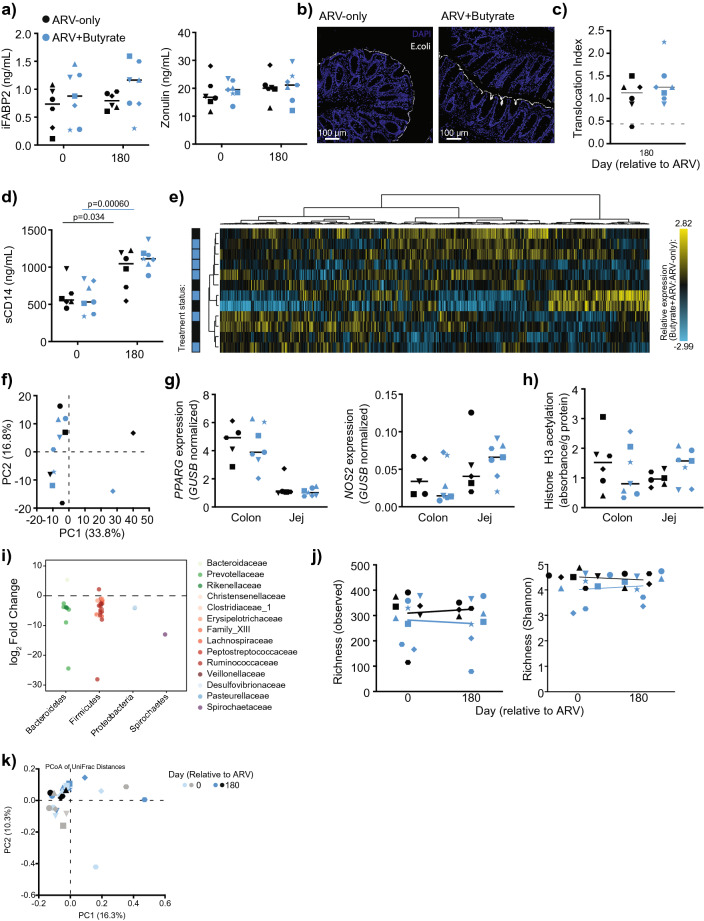
Butyrate supplementation of ARV treatment neither promotes intestinal repair nor reduces microbial translocation. (**a**) Plasma iFABP2 (left) and zonulin (right) concentrations in ARV-only (black) and ARV + butyrate (blue) animals, with individual animals denoted by individual symbols as identified in Table 1. (**b**) Representative IHC images of day 180 colons stained for *E. coli* from an ARV-only (left) or ARV + butyrate (right) animal. (**c**) Translocation index of Colon sections stained for *E. coli* as in (**b**). Dashed line represents the colonic translocation index of a single, healthy macaque stained as a batch control. (**d**) Plasma sCD14 concentrations. (**e**) Heatmap depicting relative colonic transcript abundance (columns) in macaques (rows) at day 180 as assessed by NanoString. Animal treatment status (left, as in **a**) and relative expression values (right), as indicated. Data clustered by Euclidian distance with Ward's minimum distance linkage. (**f**) PCA considering treatment-group and within-animal relative frequency of Colonic transcripts at day 180 as assessed in (**e**). (**g**) Relative expression of *PPARG* and *NOS2* in the Colon and Jej, at day 180 as assessed in (**e**). (**h**) Normalized histone H3 acetylation expression as measured by ELISA in the Colon and Jej at day 180. (**i**) Fold-change frequencies of ASVs identified as differentially abundant in response to butyrate supplementation. Data show ARV + butyrate:ARV-only frequency fold-change, color-grouped by phylum and individually shaded by family as indicated. (**j**, **k**) Alpha (**j**) and Beta (**k**) diversity estimates at days 0 and 180. Alpha diversity estimates include observed (left) and Shannon (right) richness. Beta diversity assessed by unweighted UniFrac. All timepoints are relative to the day of ARV initiation. Lines among *p* values span timepoints considered significantly different, color-coded by treatment status. Solid lines among datapoints represent means. Dashed lines among datapoints represent a SIV-batch control. Significance methods as follows: unpaired and paired two-way *t*-tests as appropriate (**a**, **c**–**d**, **g**–**h**, and **j**), MetaLonDa (**i**), and adonis (**k**).

In HIV and SIV infection, epithelial damage and microbial translocation are evident and only partially mitigated by ARV therapy^3,4,6^. As SCFAs promote colonic epithelial integrity^12^ we next determined whether butyrate supplementation altered biomarkers of epithelial integrity in the context of ARV treatment. Measured by enzyme-linked immunosorbent assay (ELISA), no significant differences in circulating intestinal fatty-acid binding protein 2 (iFABP2) nor zonulin were observed for either group from baseline to day 180 post-treatment; we did not observe a difference between groups at either timepoint (Fig. 4a). To determine whether butyrate supplementation ameliorated microbial translocation, we next assessed the presence of *Escherichia coli* in colonic tissue by immunohistochemistry (IHC) at day 180 post-treatment. *E. coli* was evident in colonic tissues at levels above that observed for a healthy, uninfected batch control; however, no significant differences in translocation were evident between ARV-only and ARV + butyrate animals (Fig. 4b, c). As microbial translocation is not limited to *E. coli*^7,42,43^, we further assessed circulating levels of sCD14 as a surrogate biomarker of microbial translocation^44^. We saw a surprising increase in sCD14 for both groups from baseline to day 180; however, sCD14 levels were comparable between groups at both assessed timepoints (Fig. 4d).

In part, butyrate supports epithelial integrity and intestinal immunity by transcriptional control, both directly through its interaction with PPARg^45^ and indirectly by global inhibition of histone H3 deacetylase^15–18^. To assess whether butyrate supplementation altered the colonic transcriptional profile, we quantified 770 immunological transcripts by NanoString^46^. No differences in expression profile were evident between groups at day 180 post-treatment (Fig. 4e) and transcriptional profiles did not cluster together by group by Ward's linkage nor by principal component analysis (PCA; Fig. 4f). Transcripts mapped to immunological pathways by ingenuity pathway analysis (IPA) as expected, but there were no differences in activation status (z-score) between the two groups (Supplementary Table S1). We similarly did not see any differences among jejunal transcripts (Supplementary Fig. S1 and Supplementary Table S1).

Despite the absence of effect on immunological transcript expression, we sought to determine whether butyrate supplementation was able to effectively modulate either specific transcriptional regulation or global epigenetic reprogramming in the context of treated, chronic SIV infection. In its role as a regulator of b-oxidation and intestinal hypoxia, butyrate is known to signal through PPARg, leading to the altered expression of nitric oxide synthase 2 (*NOS2*)^14^. No differences in either *PPARG* or *NOS2* were observed between ARV-only and ARV + butyrate animals in either colonic or jejunal homogenates at day 180 post-treatment (Fig. 4g). As expected, given the absence of other effects in our animals, no differences in histone H3 acetylation were observed in either colonic or jejunal homogenates at day 180 post-treatment (Fig. 4h).

In therapeutic models of murine colitis, butyrate supplementation enhances inflammation resistance and is accompanied by modest shifts in the intestinal microbiome^47,48^. To investigate whether butyrate supplementation might influence the microbiome in the context of ARV-treated chronic SIV infection, we characterized the fecal microbiome by 16S Illumina sequencing and identified longitudinal amplicon sequence variant (ASV) perturbations utilizing MetaLonDA. Several ASVs declined over the treatment period, concentrated particularly among Bacteroidetes *Prevotellaceae* and Firmicutes *Ruminococcaceae* (Fig. 4i). These isolated differences did not translate into a difference in either alpha or beta diversity at day 180 post-treatment (Fig. 4j, k). Collectively, our findings indicate that oral butyrate supplementation does not improve upon ARV-mediated immune reconstitution or inflammation reduction.

## Discussion

ARV therapy has significantly improved the lifespan and quality of life for people living with HIV and yet, prolonged treatment is not without complication. Non-AIDS related co-morbidities are now a leading cause of mortality for people living with HIV and are attributed to inflammation^1^ stemming, in part, from deficiencies in gastrointestinal immunity and epithelial integrity^49^. Microbiome-derived SCFAs such as butyrate are essential for the development and maintenance of gastrointestinal immunity and epithelial integrity^12,13^. Butyrate-producing bacteria are reported at lower frequencies in people living with HIV regardless of treatment status^26–34^ and the loss of butyrate production and butyrate-producing genes correlates with residual inflammation^31,37^. Although the use of prebiotic and probiotic therapies improves immune reconstitution and reduces inflammation in treated people living with HIV^31,50^ and infected macaques^35,36^, it is unclear whether butyrate directly contributes to these outcomes. Herein, we demonstrate that oral butyrate supplementation in ARV-treated macaques is insufficient to improve immune reconstitution and epithelial integrity or to reduce microbial translocation.

Butyrate supplementation has been shown to prevent or reverse inflammation in murine models of intestinal bacterial infection and colitis^47,48,51^. Our data suggest that the benefits of prebiotic and probiotic taxa in treated lentiviral infections^31,35,36^ may be multi-factorial and/or independent of butyrate activity. In addition to SCFAs, probiotic taxa synthesize many metabolites useful for host immunity and epithelial integrity and additionally compete with pathobionts for intestinal niches^12,13^. These functions may be needed to supplement the activity of butyrate. Conversely, the short-term use of antibiotics known to deplete butyrate-producing taxa—clinically indicated for the emergence of secondary and opportunistic infections—does not significantly worsen clinical indices of inflammation in treated people living with HIV^52^ nor in untreated macaques^10^ which may suggest that butyrate production is saturated throughout progressive lentiviral infections. Importantly, frequencies of butyrate-producing bacteria alone do not dictate net butyrate production, with the presence/absence of non-butyrate producing taxa directly contributing to variability in butyrate concentrations and saturation thresholds in vitro^53^. These complex interactions may limit the efficacy of monotypic therapeutics. Our work did not address the necessity of butyrate signaling in contributing to immune reconstitution in either ARV treatment alone or in the context of probiotic therapeutics. Another possibility is that absorbance of butyrate administered orally limited its potential effectiveness within the large intestine.

Butyrate influences intestinal immunity by two predominant mechanisms—signaling through PPARγ to drive β-oxidation^14^ and the inhibition of HDACs to epigenetically regulate transcriptional programming^15–18^. We did not observe a butyrate-effect in either the intestinal transcriptome, *PPARG* or *NOS2*, or in H3 acetylation (Fig. 4). Although lentiviral infections have been shown to specifically alter these parameters^54–57^, disruptions to butyrate-responsive pathways may not be the primary roadblock to complete immune restoration. Lentiviral infections induce widespread epigenetic changes^58,59^, many of which persist or rise anew after the initiation of ARV therapy^60–63^. Although HDAC inhibitors have been successfully used to reactivate viral reservoirs in latency reversal studies^64–66^, sodium butyrate treatment was not associated with increased viremia in our study (Fig. 1). The timing of ARV administration may also contribute to unique reconstitution hurdles. Early ARV initiation can limit many complications, including immune activation, inflammation, CD4+ T-cell loss, and gastrointestinal damage^3,67^; however, perturbations in the intestinal microbiome and microbial translocation remain evident^37,68,69^.

The administration of sodium butyrate in healthy mice is associated with a significant shift in beta diversity, with some evidence for a loss of butyrate-producing species^47,48^. Although the predominant loss of *Ruminococacceae* ASVs in our butyrate-supplemented animals (Fig. 4i) complements these previous observations, we did not observe significant shifts in beta diversity (Fig. 4j). In addition to an absence of immunological perturbations (Figs. 1, 2, 3, 4), these findings suggest that there remains an impediment to butyrate signaling in ARV-treated, SIV-infected non-human primates which cannot be overcome by supplementation alone. This impediment may originate from within the microbiome itself, with ratios of butyrate producers to non-producers contributing to butyrate saturation thresholds^53^. However, as butyrate-enhanced reductions in intestinal permeability and microbial translocation are at least partially independent of the microbiome^48^—directed largely through HIF1a (hypoxia-inducible factor 1-alpha) and PPARg signaling^14,48^—non-efficacy in our supplemented animals may reflect an uncharacterized disruption in these host-intrinsic pathways. Lastly, there remains the possibility that sodium butyrate was unable to reach sufficient concentrations within the intestinal lumen of our ARV-treated macaques, which could be remedied in future studies with the use of the butyrate prodrug tributyrin^70^.

In summary, we demonstrate that sodium butyrate supplementation of ARV therapy in chronically SIV-infected macaques is insufficient to improve immune reconstitution. Butyrate treatment was well-tolerated but unable to alter cellular and transcriptional measures of immunity and did not suppress microbial translocation. Further work is needed to determine the utility of supplemental butyrate to improved outcomes in the context of ARV therapy, with and without adjunct probiotics.

## Methods

### Animals and vancomycin treatment

Study design and data reporting in this manuscript follows the recommendations in the (ARRIVE) guidelines. Thirteen SIV-infected male rhesus macaques (*Macaca mulatta)*, aged 6–15, were assigned to an ARV-only (n = 6) or ARV + Butyrate treatment (n = 7) group as in Table 1, with sample size based on previous studies of experimental manipulations of disease progression in the macaque model. Groups were stratified by weight and genotype (Mamu-A*001, -A*002, -B*008, and -B*017) and animals sampled as mixed populations. All animals were infected with SIV_mac239X_ for greater than 136 days. Animals were treated with 1 mL/kg/day s.c. of emtricitabine (40 mg/kg; Hangzhou API Chem), tenofovir disoproxil fumarate (5.1 mg/kg; Hangzhou API Chem), and dolutegravir sodium (2.5 mg/kg; Hangzhou API Chem) solubilized in 15% (w/w) Kleptose (Roquette) in water^71^. Two weeks after ARV treatment initiation, ARV + Butyrate animals received sodium butyrate (Bodybio) as a single oral tablet (600 mg) mixed with food treats. Dose was chosen based on the commercial availability of over-the-counter tablets within the desired range of 500–1000 mg/day—a range which corresponds to 50–100% of the lowest daily demand reported in the adult intestinal lumen^72^. Respective treatments were continued throughout the duration of the study. The NIAID Division of Intramural Research Animal Care and Use Program, as part of the NIH Intramural Research Program, approved all experimental procedures (animal study protocol LVD 26). The Program complies with all applicable provisions of the Animal Welfare Act and other federal statutes and regulations relating to animals.

Animals were housed and cared for at the NIH Animal Center, under the supervision of the Association for the Assessment and Accreditation of Laboratory Animal Care (AAALAC)-accredited Division of Veterinary Resources and as recommended by the Office of Animal Care and Use Nonhuman Primate Management Plan. Husbandry and care met the standards set forth by the Animal Welfare Act, Animal Welfare Regulations, as well as The Guide for the Care and Use of Laboratory Animals (8th Edition). The physical conditions of the animals were monitored daily. Animals in this study were exempt from contact social housing due to scientific justification, per institutional animal care and use committee (IACUC) protocols, and were housed in non-contact, social housing where primary enclosures consisted of stainless-steel primate caging. Animals were provided continuous access to water and offered commercial monkey biscuits twice daily as well as fresh produce, eggs and bread products twice weekly and a foraging mix consisting of raisins, nuts and rice thrice weekly. Enrichment to stimulate foraging and play activity was provided in the form of food puzzles, toys, cage furniture, and mirrors or television.

### Plasma viral RNA

Viral load assessed as previously described^10^.

### Sample collection

Samples were collected as previously published^10,36,55,73^. Blood, BAL, stool, and biopsies from colon, mesenteric lymph nodes, jejunum, and rectum were collected longitudinally or at necropsy (day 180 post-treatment). Sampling occurred in random order. Neither the investigators nor the animal handlers were blinded to group allocation to ensure multi-lateral supervision of design and palliative treatment. Animals were sedated with Ketamine HCl at 10 mg/kg intramuscular (i.m.) for longitudinal blood sampling or with Telazol at 3–4 mg/kg i.m. for tissue timepoints. For jejunal biopsies, animals were further anesthetized with isoflurane gas by intubation, to effect. Successful anesthetization was monitored by response to stimuli.

Euthanasia was initiated at experimental endpoint (day 180 post-treatment) using protocols consistent with the American Veterinary Medical Association (AVMA) guidelines. Animals were first sedated with Telazol at 4 mg/kg i.m., followed by Pentobarbital at 80 mg/kg to achieve euthanasia. No animals met clinical endpoint criteria as defined by: (a) loss of 25% body weight from baseline weight when assigned to the protocol, (b) major organ failure or medical conditions unresponsive to treatment, (c) complete anorexia for 4 days or an inability to feed or drink sufficient nutrients to maintain body weight without assistance for 7 days, (d) distress vocalization unresponsive to treatment or intervention for 7 days, or (e) tumors arising from other than experimental means that grew in excess of 10% of body weight, impaired movement, or ulcerated.

For BAL collection, silicone tubing was directed into the trachea with the assistance of a laryngoscope, whereupon warmed normal saline was instilled and subsequently aspirated for collection. For longitudinal RBs, fecal material was removed from the rectum and biopsies obtained with biopsy forceps. Longitudinal MLN biopsies were obtained by laparoscopy and longitudinal jejunal biopsies by video-guided endoscopy, with samples collected by biopsy forceps. 10 intestinal pinch biopsies were obtained per animal for longitudinal assessments. Biopsies collected at necropsy were directly accessed immediately post-mortem. Biopsies were maintained in RPMI prior to processing.

Approximately 1 mL of macaque stool was collected fresh from each animal by inserting a sterile swab 2 cm into the rectum and spinning to collect available sample. Collected feces were snap-frozen and stored at − 80 °C until accession.

Biopsies were maintained in RPMI-1640 medium for transport and rinsed twice with PBS prior to processing. Plasma was isolated from blood by centrifugation. Mononuclear cells were isolated from blood by Ficoll gradient centrifugation and from BAL and tissue biopsies by straining/grinding samples through a 0.22 μm cell strainer. Three of 10 intestinal pinch biopsies were transferred to soil-grinding Precellys tubes (Bertin Technologies, France) and homogenized in 1 mL TRIzol (ThermoFisher Scientific, USA) at room temperature on a Precellys 24 homogenizer at 5000 revolutions per minute (RPM) in 4 successive 20 s intervals. TRIzol-preserved homogenates were immediately transferred to − 80 °C for storage.

### Immune phenotyping and functional assessment

Polychromatic flow cytometry and cell sorting were performed on stained mononuclear cells as previously described^36^, using a BD LSRII (FACSDiva v9.0). Cell were stimulated for functional assessment by overnight culture with phorbol myristate acetate (2.5 ng/mL) and ionomycin (1 μg/mL), in the presence of brefeldin A (1 μg/mL). Antibodies against the following antigens were used for staining at predetermined concentrations: CCR5 (clone 3A9), CD20 (2H7), CD28 (CD28.2), CD3 (SP34-2), CD45 (D058-1283), CD69 (FN50), Ki-67 (B56), and TNFa (MAb11) from BD; CD103 (BLy7), CD4 (OKT4), CD8 (SK1), IFNg (e450), and IL-17 (eBio64DEC17) from Thermo Fisher-Scientific; CD95 (DX2) and IL-2 (MQ1-17H12) from Biolegend; and FOXP3 (3G3) from Miltenyi.

Cell viability was assessed using the Live/Dead Aqua Fixable Dead Cell Stain (Thermo Fisher-Scientific). CD4+ and CD8 + TM were defined as CD95 + singlet, clean, live, CD3 + lymphocytes with positive/negative gating based on clearly grouped populations, historical-determined expression, and the use of internal controls (Supplementary Fig. S2). A threshold of 100 collected events in the parent population was utilized for all subset expression analyses (FlowJo 9.9.6).

### ELISAs

Concentrations of iFABP2, zonulin, and sCD14 were quantified from plasma and acetylated histone H3 was quantified from colon intestinal homogenate histone extracts using commercially available ELISA kits (My Biosource #MBS740424, Alpco #30-ZONSHU-E1, R&D #DC140, and abcam #ab115102, respectively), according to the manufacturers’ protocols. Histone extracts were normalized to 200–400 ng/uL by BCA protein assay (Millipore). All samples were assessed as technical duplicates. iFABP2 and sCD14 were independently confirmed (data not shown).

### RNA extraction, quantification, and assessment

For intestinal transcript quantification TRIzol-preserved samples were thawed and treated with 200 μL chloroform to separate nucleic acid into an aqueous phase. Following separation, Total RNA was isolated from the aqueous phase using the MagMAX-96 total RNA isolation kit (ThermoFisher Scientific) per the manufacturer's protocol. RNA concentration and purity (A260/280 ≥ 1.8) were assessed by spectrophotometer prior to cDNA generation and normalized to 50–100 ng/μL in PCR-grade H_2_O. For transcript quantification by NanoString, preparation, hybridization, and detection of RNA samples were carried out by following the NanoString manufacturer’s instructions (NanoString Technologies) using the nCounter NHP Immunology Panel. Subsequent analyses were performed using the nSolver (v4.0.70) analysis system (NanoString Technologies). Reads from NanoString were normalized to internal positive and negative controls and housekeeping genes. For visualization by heatmap, transcript data were clustered by Euclidian distance with Ward's minimum distance linkage. Transcriptome variability was visualized by PCA using the ggbiplot package (v0.55) in RStudio (v1.1.463) using R (v3.6.2). NanoString quantified transcripts were further ascribed to canonical gene expression pathways by IPA (Qiagen v01-19-02). The relative expression of individual transcripts *PPARG and NOS2* was assessed from the NanoString generated counts, normalized to endogenous glucuronidase beta (*GUSB*)*.*

### 16S isolation and analysis

16S rDNA was isolated and sequenced as previously described^73^. Raw Illumina FASTQ files were first demultiplexed using a custom Python script. Returned paired-end FASTQ reads were filtered and processed using the DADA2 package (v1.14.1) in RStudio using R to infer ASVs at a 99% identity threshold using the Silva database (v132). Before quality trimming 3,050,651 reads were included in 26 samples with an average of 117,333 reads per sample. Reads were trimmed to 225 bp (forward) or 200 bp (reverse) and filtered to exclude sequences with degenerate bases (N), more than 2 expected errors (maxEE), or chimerism. DADA2 quality trimming resulted in 2,056,542 reads for all the samples with an average of 79,098 reads per sample. 2 samples with less than 1000 reads were omitted from further analysis. ASVs identified as non-Bacteria, mitochondria (Rickettsiales *Mitochondria*), and Cyanobacteria were removed from further consideration as were resultant genera at less than 3% prevalence or phyla with no genera diversity. Longitudinally, differentially abundant taxa were identified by MetaLonDa (v1.1.8) in RStudio using R. Fully analyzed 16S miSeq data (n = 24) are deposited in the NCBI Sequence Read Archive (SRA) under project number PRJNA779879.

### Immunohistochemistry

Five µm paraffin sections were incubated at 60 °C for 60 min, dewaxed in xylene and rehydrated in ethanol. Ag retrieval was performed in pH 6.0 0.00356 M citric acid solution in a steamer for 45 min. Slides were blocked in Intercept^®^ (PBS) Blocking Buffer (LI-COR). Slides were stained with polyclonal Rabbit Anti-*E. coli* (Dako #B0357) and Spectral DAPI (AKOYA Biosciences). Images were acquired on a Leica SP8 inverted confocal microscope equipped with HyD hybrid detectors. Between 1 and 2 stained tissue slides of each sample were scored blindly by 4 individuals on a scale of 0 (no *E. coli* present) to 3 (present throughout the sample). Reported scores reflect the mean scores of both slides and individual scorers.

### Statistical analyses

Longitudinal viral load was assessed by two-way ANOVA with Bonferroni’s multiple-comparison test (Prism v9.0, GraphPad Software Inc.). T-cell phenotype and function, ELISA analyte concentration, translocation index, individual transcript expression, histone H3 acetylation levels, and alpha diversity indices were assessed by paired and unpaired, two-way *t*-tests (Prism), as appropriate. Bulk transcript expression acquired by Nanostring was assessed within nSolver by *t*-test and included the Benjamini–Yekutieli false discovery rate. IPA canonical pathway enrichment and z-score determined by IPA using default parameters. Longitudinally, differentially abundant bacterial ASVs were identified by MetaLonDA. Differences in unweighted Unifrac beta-diversity were assessed by the adonis function in RStudio using R. No data that met minimum threshold requirements as outlined in the Methods were excluded.

## Supplementary Information

Supplementary Information 1.

Supplementary Information 2.

## Data Availability

16S MiSeq data are deposited in the NCBI SRA under Project No. PRJNA779879. Other data available from this study are available from the corresponding author upon reasonable request.
