# Supplementary material for: Butyrate administration is not sufficient to improve immune reconstitution in antiretroviral-treated SIV-infected macaques
Source: Sci Rep. 2022 May 6;12:7491. doi: 10.1038/s41598-022-11122-x (PMC9076870; doi:10.1038/s41598-022-11122-x)
Supplement: Supplementary file 1 — Supplementary Information 1. [file 41598_2022_11122_MOESM1_ESM.docx]

**Supplementary Figure 1. Butyrate supplementation of ARV does not alter Jejunal immunological transcript expression.**

(**a**) Heatmap depicting relative jejunal transcript abundance (columns) in macaques (rows) at Day 180 post-treatment as assessed by Nanostring. Animal treatment status (left) and relative expression values (right), as indicated. Data clustered by Euclidian distance with Ward's minimum distance linkage. (**b**) PCA considering treatment-group and within-animal relative frequency of jejunal transcripts at Day 180 post-treatment as assessed in **a**. Individual animals are denoted by individual symbols as identified in Table 1.

 **Supplementary Figure 2. Representative flow cytometry gating and staining.**

(**a**) Representative gating and staining showing derivation of CD4+ and CD8+ T-cells. (**b**) Representative gating and staining of assessed T-cell phenotypic and functional markers.
